# From Kyiv to Vienna: Soviet gerontology’s international influence

**DOI:** 10.1177/09526951251383670

**Published:** 2026-01-19

**Authors:** Isaac McKean Scarborough

**Affiliations:** 1 4496 Leiden University, the Netherlands

**Keywords:** geriatric medicine, gerontology, Soviet Union, United Nations, World Health Organization

## Abstract

This article tracks the influence of the Soviet school of gerontology and geriatric medicine on international science and medical practice during the second half of the 20^th^ century. Beginning with the initial development of this school of gerontology in the 1930s, this article shows how the Soviet influence on international ideas of ageing, geriatric medicine, and biological gerontology was significant, especially through the institutional scientific-medical networks established by the United Nations and the World Health Organization. This influence, moreover, was based on work conducted over the decades by the Kyiv-based Institute of Gerontology, which established itself as the centre of Soviet research on ageing and its application to medical and social care for older persons. By considering how the doctors and scientists in Kyiv were able to access international scientific networks and influence global gerontological discourses in the 1960s–1980s, this paper shows the enduring influence of Soviet concepts of biological ageing and related medical practice.

## Prelude: The World Assembly on Ageing

The World Assembly on Ageing, held in Vienna, Austria in July–August 1982, was not an obviously *Soviet* event. Its chairman, William Kerrigan, was American, and the Assembly itself was organised under the auspices of the United Nations (UN), an organisation with which the Soviet Union had at best a contested relationship.^1^ The discussions, debates, and resolutions of the Assembly, moreover, avoided any language of class, working labour, international struggle, or other rhetorical hallmarks of Soviet influence, instead emphasising the universal nature of ageing. Focus remained on the stages of the human ageing process, methods of intervention and the prevention of diseases common in older age, and the broader treatment of the growing older populations in many developed and developing countries. This was undeniably a worldwide challenge. As the World Assembly highlighted, with life expectancy rising and birth rates declining across the world, the proportion of older persons (defined as those over 60) had already reached 9% of the global population. By 2000, this was expected to rise to 10–11%, and by 2025, to nearly 14%.^2^ With life expectancy having already reached an average of 75 in some countries, moreover, there was a broad consensus amongst world governments that important conversations needed to be had about how to study, provide for, and support this growing older population.^3^

Just beside the chairman’s podium and behind the scenes, however, the Assembly was marked by a subtle but notable Soviet character. The deputy chairman of the Assembly, who answered for much of the event’s day-to-day organisation, was Vladislav Bezrukov, the head of the Experimental Division of the Kyiv-based Institute of Gerontology, the USSR’s leading research institute in the field of ageing. The Director of the Institute of Gerontology, Dmitry Chebotaryov, moreover, although formally participating in the Assembly as the head of the Ukrainian delegation, had in fact been a driving force behind its inception and development. It had been Chebotaryov who, at the 1972 9^th^ Congress of the International Association of Gerontology (IAG) in Kyiv, had discussed the idea of a World Assembly on Ageing, and who, over the following decade, had coordinated with the UN’s Ageing Unit in Vienna and the European office of the World Health Organization (WHO) in Copenhagen to bring the Assembly into reality. This influence, moreover, was visible in the final resolutions of the Assembly, from the ‘stages of ageing’ it referenced to the practices of old age care it recommended, to the broader frameworks of integrated social, environmental, and medical care it espoused, reflecting both underlying Soviet theories of ageing and guidelines produced earlier in Kyiv.

## The history of gerontology, reconsidered

The 1982 World Assembly on Ageing demonstrated the USSR’s close engagement with the global development of international research and advocacy on ageing. In fact, as the research, medical practice, and coordination with international organisations conducted in Kyiv and described in this paper show, the influence of Soviet gerontology on the development of the field was significant. This influence, however, has been largely overlooked in the existing history of gerontology. As many authors (e.g. Achenbaum, 1995; Evans, 1996) have noted, gerontology ‘came of age’ – developed and coalesced as a coherent field of scientific study – over the course of the 20^th^ century. Combining elements of the biological study of human ageing, medical research into human lifespans along with interventions to treat and/or stay those ailments more common in older age, and socially oriented research considering the treatment and support for older persons, gerontology has since the beginning of the 1900s dealt with the challenge of interdisciplinarity and the need to integrate many scientific perspectives. Paralleling its subject matter, the *history* of gerontology that has come of age over the past 30 years has equivalently faced the difficulty of interconnecting multiple scientific disciplines, geographies, and time frames. As W. Andrew Achenbaum (1995) has narrated, for example, leading American figures in the study of ageing, such as Nathan Shock at the Baltimore Longitudinal Study of Ageing (BLSA), balanced between studying the processes of aging on a biological level and proposing medical care for older persons. As Shock and others attempted to integrate these demands, so too did Achenbaum, as well as Hyung Wook Park (2016), who argues that in the United States (US), the combination of different disciplinary perspectives under the financial support of the US government was able to create the beginnings of a coherent combined school of gerontology, one that considered both the underlying processes of ageing and the experiences, medical and social, of later life. In the United Kingdom (UK), as a few authors (Evans, 1997; Park, 2009) have noted, the division between state-funded medical services and less-supported biological research created a greater split between the two disciplines within British gerontology in the post-war period.

Notwithstanding the differences in their development paths, however, in both the UK and the US a consistent theoretical approach to ageing came together by the 1970s and 1980s. This was a ‘biomedical model’ that ‘defined old age as a process of basic, inevitable, relatively immutable biological phenomena’, as Carroll L. Estes and Elizabeth A. Binney wrote in 1989 (588). This biomedical model acknowledged the presence of social and environmental factors but posited that they were far less important than individual processes of ailment and aging, which were presumed to be predicated on genes, medical histories, and personal physiological conditions. As Estes and Binney argued, this ‘trend towards individualization as one form of reductionism’ (ibid.) was based on standard practices and theories of biomedicine, which ‘attempt to explain phenomena by isolating the smallest possible units of analysis’ (ibid). In the case of ageing, this was the individual body and its process of degradation over time, not its interactions with other bodies, society, or a physical environment. As a result of this reductionism, Estes and Binney wrote, the biomedical model dominant in Anglo-American gerontology in the 1970s and 1980s tended towards ‘equating old age with illness’ (ibid.).

The dominance of the biomedical model in late 20^th^-century gerontology has been shown in studies on the later decades of the BLSA (Moreira and Palladino, 2011; Park, 2013), where population studies needed to be justified on the basis of their application to individualised physiological research. As Nathan Shock himself wrote after decades of work on the BLSA, the purpose of the study was to distinguish the ‘true effects of aging’, such as genetics or cellular degeneration, from social and environmental factors that ‘are biologically irrelevant to the underlying mechanisms of human ageing’ (Shock *et al.*, 1984: 1). The importance of this biomedical reductionism during the latter part of the 20^th^ century has further been noted by studies of British research on ageing (Moreira, 2019) and geriatric medicine in the US and UK alike (Butler, 2008; Morley, 2004; Mulley, 2012). Later, it also engendered pushback amongst advocacy groups such as the American Association of Retired Persons (AARP), which, as James Chappel (2024) has recently written, tried to reframe its members' ‘golden years’ as a time of leisure and enjoyment, not only decline and disease. The move to question the biomedical model also found later reflection in a body of critical gerontology from the 1990s, which suggested that the Anglo-American biomedical model had come to restrict the lives of older persons not only in the West (e.g. Cole, 1992; Thane, 2000) but also in the developing world (Cohen, 1992; Katz, 1996; Sivaramakrishnan, 2018). As Kavita Sivaramakrishnan has forcefully argued, the biomedical reductionism of modern gerontology, when applied to societies outside of the West, tends to disregard local conditions, norms, and histories, instead assuming that all individuals can be reduced to the same physiological standards of ageing and medical decline.

In recent years, the criticism initially voiced by Estes and Binney and later highlighted by Sivaramakrishnan and others has seemed to have an effect: biomedical gerontologists have begun to explicitly incorporate social environments and other collective factors into their models of ageing. In 2019, the preeminent gerontologist and neurobiologist Caleb Finch was quoted stating that he was newly engaged in studying ‘the environmental side of ageing’, which was something ‘that very few of my peers or colleagues in biomedical gerontology are paying any attention’. This had been ignored, Finch argued, because it had been opposed to the ‘classical reductionism of biochemistry’, but ‘this is where the frontier is’ in modern gerontology (cited in Armstrong, 2019: 211). This reflects further work reconsidering the dominance of reductionism in gerontological research, from strictly biomedical works to well-received documentary series popularising the idea of ‘Blue Zones’ where longevity has been promoted through diet, exercise, and socialisation (Jeter, 2023). Even as the models under discussion have heterogenised, however, what has remained consistent across the history of gerontology is its limited geography. A bipolar view of the scientific world is presented, with the Anglo-American school of gerontology being dominant, and the developing world pushing back. Alternative centres of gerontological research or theoretical work are not considered relevant or of influence. This bipolar divide, however, overlooks important historical actors, including, as this article will argue, those working for the Soviet Institute of Gerontology in Kyiv.

While overlooked in global histories of ageing and gerontology, important work has been done to situate Soviet gerontology in its own context. Susan Grant has written about the work conducted at the Institute of Gerontology on active ageing and the connections between healthy older age and socialist ideology (2022), as well as the design and construction of care homes for older persons in the Soviet context (2023). In line with the focus on ideology and social care, studies by Danielle Leavitt-Quist (2023) and Klots and Romashova (2023) have further pointed to the changing role of older persons in the post-Stalinist period: as the Soviet state’s focus on individual welfare increased, so did both its investment in pensions and its acceptance of older persons’ potential role in society (on Soviet pensions, also see Lovell, 2003; Mücke, 2013; Smith, 2015). Distinct from this body of literature, another has focused on the biological promises of gerontology as a field of possible ‘miracles’, highlighting the links between the Soviet Union’s transcendental view of science and the ideas of life extension held by the study of ageing (Bezrukov and Duplenko, 2023; Duplenko, 1985; Kremenstov, 2011; Tutorskaya, 2023). In both cases, however, the history of Soviet gerontology has so far remained distinct and unconnected to the history of the global discipline, without clear connection drawn between its institutions and ideas and the field’s international trajectory.

Building on this literature, however, this article attempts to problematise the current bipolar history of international gerontology. Rather than existing in a scientific vacuum or having no influence on international gerontology, the article argues, Soviet gerontology developed in close coordination with partner institutions in the US, the UK, Geneva, and elsewhere. Further, on the basis of the dominant scientific philosophy of the USSR, dialectical materialism (Graham, 1987), Soviet gerontologists developed, espoused, and spread a distinctly Soviet theory of ageing, which came to influence international discourse and practice through the UN’s and WHO’s programmes on ageing, of which the Soviet Institute of Gerontology was a founding and centrally important member. This theory of ageing, moreover, stood in contrast to the biomedical model dominant in the West in the 1970s and 1980s: it was expressly non-reductionist and emphasised both the interactions between a person and his or her environment and the fundamental malleability of physiological states. This theory, as well as some of the practical recommendations made by the Institute on its basis, this article argues, can then be observed in documents of the World Assembly on Aging in 1982 and other organisations thereafter.

To show this process of development, this article proceeds as follows. First, it outlines the institutional history of Soviet gerontology in Kyiv before turning to the establishment of links between the Soviet Institute of Gerontology and its many international partners. Over the decades between the 1950s and 1970s, these links grew stronger and more significant, as did the standing of Soviet gerontology and individual Soviet gerontologists in world circles. At the same time, Soviet gerontologists were developing a clear vision of what older age could constitute in the socialist context, and what a Soviet theory of older age would entail. As international engagement continued into the 1970s and 1980s – and as the WHO and UN increasingly turned to Kyiv as a source of expertise – as this article details, these theoretical ideas began to permeate into the international realm and into the policy documents of international organisations, predicating their recent reemergence as Western gerontologists’ ‘new’ discoveries of distinctly dialectical elements of ageing, such as environment, nutrition, and socialisation.

## Kyiv as the centre of Soviet gerontology

Twenty years before the founding of the USSR’s Institute of Gerontology, Kyiv played host to one of the world’s first national conferences on ageing. Organised by Aleksandr Bogomolets, a polymath later known for his somewhat over-hyped ‘anti-reticular cytotoxic serum’, this conference brought to Kyiv specialists on gerontology, geriatrics, and the human life cycle from across the USSR.^4^ Held on 17–19 December 1938, the conference was focused on ‘the origins of ageing and the development of mechanisms for the prophylactic treatment of its premature arrival’ (Bogomolets, 1939: 5). Building on work conducted by Bogomolets in Kyiv, as well as Aleksandr Nagornyi in Kharkov, the conference was meant to solidify Soviet Ukraine’s position as a centre of gerontological research.^5^ This harked back to the foundational work of Ilya Mechnikov, the Kharkov-born cytologist whose research in Paris in the late 19^th^ and early 20^th^ centuries had created the basis for a great deal of modern gerontology (Bogomolets, 1940: 27–33). The conference proceedings demonstrated the engagement of Soviet gerontology with broader global trends, in content largely paralleling contemporaneous conferences such as that held by Edmond Cowdry in Woods Hole, Massachusetts in 1937 (Freeman, 1973; Scarborough, 2022: 1253–4).

Bogomolets’s organisational efforts were interrupted after 1939 by the onset of World War II, as well as the post-war triumph of Trofim Lysenko’s isolationist vision of ‘Lymarkian’ genetics. Intentionally and explicitly cutting off engagement with worldwide biological and medical sciences, Soviet state support for Lysenko’s theories of non-hereditary acquired attributes also limited gerontology’s advancement (Joravsky, 1986; Kremenstov, 1997: 158–83; Medvedev, 1993). Those gerontologists who continued to advocate for and promote cellular and genetic explanations for ageing, such as Zhores Medvedev in Moscow, found support for their ideas internationally – and closed doors and institutional intransigence at home. The Soviet Union sent no representatives to the first three congresses of the International Association of Gerontologists (IAG; Liege, Belgium, 1950; St. Louis, US, 1951; London, UK, 1954), and generally made little effort to promote gerontology as a scientific or medical practice (Scarborough, 2022: 1253–5).

Matters changed radically in 1956. As the biologist Nikolai Dubinin (1973: 359–89, 401–2) later wrote, Nikita Khrushchev’s larger programme of destalinisation also included Lysenko’s essential demotion and the restriction of his authority over biological research.^6^ This, combined with the Soviet Union’s contemporaneous ‘thaw’ towards Western ideas and later decade of low-tension ‘détente’ in the Cold War, created ideal opportunities for the expansion of research – and its return to the global trajectories in which it had been embedded. While the earlier leaders of Soviet gerontology – Aleksandr Bogomolets and Aleksandr Nagornyi – had both passed away during the period of Lysenkoism, a new generation of scientists took advantage of the changing political winds to re-establish international links.^7^ This push was initially led by Elena Vasiukova, the director of the All-Union Institute of Experimental Endocrinology in Moscow, who organised trips to Romania’s K. Parkhon Memorial Institute of Geriatric Medicine in January 1957 and to the 4^th^ Congress of the IAG in Merano and Venice, Italy in July 1957.^8^ On both trips, however, Vasiukova brought with her Bogomolets’s former students: D. F. Chebotaryov, a Kyiv-based prenatal medicine specialist, who travelled to Romania, and F. D. Marchuk, a geriatrician who had worked extensively with Bogomolets, who accompanied her to Italy.^9^

This background network of Ukrainian specialists was actualised during the discussions that followed Vasiukova and Marchuk’s trip to Italy about the need to promote Soviet research into gerontology. In her report on the congress, Vasiukova emphasised the need to both ‘Found a scientific association of gerontologists and join the international association of gerontology … [and] found a scientific-research institute for gerontology, which would act as the organizing centre for questions of gerontology’.^10^ This resulted in the creation of a formal joint committee for gerontology and geriatrics overseen by the Ministry of Healthcare of the Soviet Union and the USSR’s Academy of Medical Sciences. In addition to representatives of many Moscow-based ministries and institutes, the committee also included Nikolai Gorev, another student of Bogomolets, who was at the time a division head in Kyiv’s Institute of Physiology.^11^ Gorev was also assigned the role of evaluating the ‘state and perspective for scientific research into gerontology and geriatrics in the USSR’.^12^ As discussions in the committee turned to the idea of creating a dedicated institute of gerontology in the USSR, Gorev and Bogomolets’ other students were able to use their positions to present Kyiv as the most appropriate location for this institute. Working in both Kyiv and Moscow – as early as February 1958, in fact, they had convinced the Ukrainian Academy of Medical Sciences to formally assign a building for the as-of-yet unfounded Institute – they were able to see off arguments favouring an institute in Moscow, Tbilisi, or Poltava.^13^ This finally led to the formal opening of the Institute of Gerontology and Experimental Pathology in Kyiv in May 1958 (Chebotarev, 2008: 62). Gorev was appointed its founding director; Chebotaryov and Marchuk were amongst his deputies.

## Soviet gerontology looks abroad (1958–1970)

From its foundation, the Institute of Gerontology positioned itself as much outwards – towards international gerontology and geriatric practice – as inwards towards Soviet society. Although the combined nature of the institute, which included both fundamental research and clinical practice under one roof, was frequently touted as a unique advantage, this in fact replicated international practice, such as the Institute of Gerontology in Bucharest or the well-known collaboration between the Bethesda City Hospitals and the US National Institute of Health’s Ageing Unit, set up by Edward Stieglitz and Nathan Shock in the 1940s (Park, 2013; Shock and Baker, 1988: 4–5). (The founders of the Institute of Gerontology in Kyiv, moreover, had a keen interest in the US’ gerontological institutions, and would pursue contacts with the National Institute of Ageing over the following decades.) By and large, the activities of the Kyiv Institute and its directors were aligned with international practices in gerontology: they emphasised both research into the biological processes of ageing, on the one hand, and the need to improve the lives of older persons through medical research and the development of geriatric medicine, on the other.

Dmitry Chebotaryov was at the forefront of efforts to internationalise the work of the Institute. A clinician and specialist in prenatal surgery known for his ‘quiet enthusiasm’ and ‘kindly attitude to old people generally’, Chebotaryov had been born in 1908 in Kyiv; he worked throughout Ukraine and southern Russia before returning to Kyiv after World War II.^14^ Importantly, he was also extremely well-connected in political circles, having by the mid-1950s reached the position of director of the Ukrainian Ministry of Health’s ‘Fourth Main Directorate’, which was in practice the medical service for Communist Party leaders.^15^ Although initially appointed deputy director and head of the clinical wing of the Institute of Gerontology in 1958, Chebotaryov was promoted to director in 1961 (Chebotarev, 2008: 65).^16^ Even before becoming director, however, he began reaching out to international partners, including the IAG, securing invitations for himself and another researcher from Kyiv, Venyamin Frol’kis, to the 5^th^ IAG Congress, held in San Francisco in 1960 (Scarborough, 2022: 1257). At the same time, the Soviet doctor Mikhail Akhmeteli was working as an advisor on chronic diseases and geriatrics in the WHO European headquarters in Copenhagen, Denmark.^17^ A tuberculosis specialist originally from Tbilisi – but who had made a career in Moscow’s research institutes before being seconded to the WHO – Akhmeteli appears to have been familiar with Chebotaryov and, in 1962, invited him to become a consultant to the WHO and to participate in discussions in Copenhagen related to international coordination of gerontological research and geriatric practice.^18^ These discussions resulted in one of the WHO’s first international symposiums on ageing, which was held on 14–22 May 1963 in Kyiv.

This ‘Seminar on the Health Protection of the Elderly and the Aged and the Prevention of Premature Ageing’ brought experts on ageing from across Western Europe to Kyiv, including the well-known gerontologists Dr Walter Doberauer from Vienna and Dr R. J. Zonneveld from Leiden. Other participants came from Belgium, Bulgaria, Hungary, Denmark, Ireland, Italy, Norway, Poland, Romania, Britain, West Germany, Finland, France, Czechoslovakia, Sweden, and Switzerland.^19^ Guided by Chebotaryov and Akhmeteli, these experts collectively developed a series of standard protocols for assessing ‘natural’ versus ‘premature’ ageing and determining a patient’s ‘biological age’. A concept that had been developed over the decades by both early Soviet and even pre-Soviet imperial thinkers, including Bogomolets and Ivan Tarkhnishvili, ‘premature ageing’ suggested that external physical, social, and environmental factors could dialectically lead to a decline in biological health more rapidly than the passage of years on their own would suggest.^20^ This aligned with the seminar’s work to also consider and outline age ranges against which such ‘premature ageing’ could be compared – and which could be used to standardise clinical notions of ‘middle age’ (45–60 years), ‘aged’ (60–75 years), ‘old’ (75–90 years), and ‘very old’ or ‘longevous’ (90+ years) people.^21^ In addition, as Chebotaryov described in his memoirs, the combination of medical practitioners and research scientists at the seminar allowed for a serious discussion about ‘Whether or not old age should be considered a disease or a biological condition’ (Chebotarev, 2008: 93). Although seemingly pedantic, this basic philosophical question, Chebotaryov pointed out, determined how older patients and persons should be treated. If ageing itself was a ‘genetically programmed set of diseased states’, he wrote, then this meant that any person ‘as they grow older, will inevitably grow ill and this illness will ultimately lead to death’. Alternatively, if ageing was a ‘physiological condition’, then, with age, an older person’s ‘body grows vulnerable, and this vulnerability abets the development of those factors that form the basis for pathologies’. In other words, ageing itself was not the source of ailment – rather, disease was simply more likely to find a foothold in older bodies. ‘This was advantageous’, Chebotaryov concluded,

because then it was possible to treat a sick person, heal their illnesses, and be assured that this was not a sign of ageing, but instead a tangential phenomenon: an infection or something else … that could be changed through lifestyle amendments, medical treatment, or physical therapy, and in this way extend the life of the ill person for a number of years. (Chebotarev, 2008: 94)

The 1963 WHO Seminar in Kyiv would prove both theoretically and institutionally indicative of the trajectory that the Institute for Gerontology would follow in the following decades. On a theoretical level, the resolutions passed by the seminar and the discussions held in the Institute demonstrated a growing consensus around a particularly Soviet and socialist view of ageing. This was one that saw ageing as a ‘physiological condition’ and not an ailment or a disease. It was also an explicitly dialectical perspective: working with the underlying Soviet philosophy of science, dialectical materialism, Soviet gerontologists posited that aging was both a materially definable process and one that was the product of many interrelated factors (on dialectical materialism in the USSR, see Graham, 1987). In direct contrast to the biological reductionism identified by Binney and Estes in the contemporary West, this was a theory of ageing that emphasised the social, ecological, and ‘constantly evolving changes of daily activity’, as Frol’kis (1973: 27) wrote, that underpinned life expectancy and long-term health outcomes (also see Berdyshev, 1978: 24; Chebotarev, 1973: 7–8). Viktor Kozlov, an anthropologist who worked closely with the Institute of Gerontology in Kyiv, later summarised the position:

It would be mistaken to consider the phenomenon of longevity to be a singularly genetic issue … Human nature is integrally social, combining in itself all levels of physical development; the highest [level], the social, subordinates to itself all lower levels, including the biological. Expressions of man’s human nature are socially intermediated and oriented in accordance with the laws of the higher social orders of physical change. (Zubov and Kozlov, 1982: 15)

According to this Soviet theory of ageing, older age was as much a product of an individual’s surroundings and activities as of biology or genetics – and was, just as importantly, something malleable, changeable, and not bound to end in disability or disease.

The Soviet perspective on dialectical ageing would influence not only theoretical works on older age and longevity, but also practical tools of diagnostic and medical care that were distributed internationally. For example, the 1963 WHO Seminar’s attempt to set clear and well-defined boundaries for age ranges was an important step towards materialising the processes of ageing and providing clear protocols for treatment at each stage of older age. Previously, the lack of agreed-upon ‘norms’ and boundaries between age groups, such as ‘elderly’ and ‘aged’ patients, had been noted as holding back international research into the diseases more common in older age.^22^ Now, however, in place of vague terms that had been potentially erroneously compared, there were WHO-approved recommendations on how to categorise periods of ageing. At the same time, though, the Seminar resolutions noted that the categories were themselves malleable: ‘These categories are significantly broad and intended for practical use, without strictly restricted limitations on the boundary between one group and another’.^23^ The point was not to lock down individuals into categories, but instead to consider the ways in which the stages of older age might both help to provide direction to medical care while also accommodating changes in social order, environment, and other factors. In practice, Soviet gerontology and geriatric medicine used this perspective to emphasise the need for socially-oriented elements of life: exercise, social engagement, diet, and work. Both popular and scientific publications from the 1960s and 1970s emphasised specific exercises for older persons (e.g. Chebotarev, 1970: 74–9), ways of remaining engaged in labour (Valentei, 1977: 6–9), and the need for active social networks in older age (Lakiza-Satchuk, 1977; Vinogradov and Revutskaya, 1972: 262–3).

Following the 1963 WHO Seminar, moreover, both the influence of the Institute of Gerontology’s ideas and its collaborative network would begin to expand. Later that year, Chebotaryov spearheaded the founding of the All-Union Society of Gerontologists and Geriatricians (*Vsesoiuznoe obshchestvo gerontologov i geriatrov*), which joined the IAG as a constituent society and correspondingly expanded the Soviet presence at IAG conferences (Chebotarev, 2008: 82–3; Shock and Baker, 1988: 89). In Copenhagen for the IAG Congress, moreover, Chebotaryov sought out meetings with some of the world’s leading gerontologists, spending a long lunch describing to Nathan Shock the Institute of Gerontology and his plans to ‘develop a research program on the basic biology of ageing’ (Shock and Baker, 1988: 95). He also encouraged Shock and others to visit Kyiv and develop joint collaborative projects.^24^ In 1965, Chebotaryov’s efforts came together in the first of a series of WHO-funded European ‘Courses on Medical and Social Aspects of Gerontology’, held in Kyiv but partially taught by leading Western gerontologists. The central figure at the 1965 course was undoubtedly William Ferguson Anderson of the University of Glasgow.^25^ The holder of the world’s first university chair in Geriatric Medicine, Anderson had done a great deal to establish geriatrics and practical gerontology in the United Kingdom (Brocklehurst, 1997; Thane, 2000: 436–58). Having developed friendly relations with Akhmeteli during the latter’s time at the WHO office in Copenhagen – he was now the deputy director of the Foreign Relations Department of the Ministry of Healthcare of the USSR – Anderson was happy to accept his invitation to lead the Kyiv-based course in 1965 (Anderson, 2007: 128).^26^

The WHO courses were intended for both Soviet and Eastern European geriatricians, and the WHO funded the participation of doctors from Romania, Czechoslovakia, Bulgaria, Poland, Hungary, and Yugoslavia.^27^ Their content (102 hours of lectures and 36 ‘practical hours’ over the course of a month) included demographics, treatment protocols for older patients, biological aspects of ageing, and an extended focus on the processes of ageing – and the diseases connected with ageing – observed in particular organs of the human body.^28^ Meant to both share best practices and experiences and familiarise socialist doctors with newly developed Western geriatric medicine, the 1965 courses were considered by Anderson (2007: 129) to have been a great success: everything was well organised and his ‘lectures were well received’. The courses also provided a successful venue for Kyiv to promote its ideas about ageing internationally: two years after the 1965 courses, Anderson (1967: 1227–8) published a supportive article in the *Proceedings of the Royal Society of Medicine*, in which he approvingly noted the newly established WHO categories of ageing, identified them as deriving from Kyiv, and suggested their flexible character was fitting in a time of expanding lifespans.

The 1965 WHO courses in Kyiv also set the stage for further international collaboration between the Institute of Gerontology in Kyiv and international partners. The courses were repeated in 1968, again with Anderson’s participation, and Chebotaryov managed to secure the additional visits of leading gerontologists to Kyiv that he had been cultivating.^29^ In early 1968, for example, both Nathan Shock and Fritz Verzar, a leading Swiss gerontologist, visited Kyiv for a symposium on biological ageing.^30^ These visits intertwined with the formal relationships between the Institute and the WHO and the IAG. As a formal ‘consultant’ to the WHO, Chebotaryov was able to frequently travel to meetings across Europe, and he encouraged members of his staff, including Vladislav Bezrukov, to improve their English language skills and participate in both foreign travel and assisting Western visitors to Kyiv.^31^ With the All-Union Society of Gerontologists now a constituent body of the IAG, moreover, Chebotaryov was able to take on formal roles in the association, increase Soviet representation at its conferences, and travel to international and regional conferences. In 1969, Chebotaryov travelled to Washington, DC, for the 8^th^ International Congress of the IAG, where he first met with Tarek Shuman, soon to become the director of the Ageing Unit of the UN Centre for Social Development and Humanitarian Affairs, then based in New York City.^32^ Within a few years, both Chebotaryov and the Institute would be listed amongst the UN’s central partners for the study of ageing in Europe.^33^

By the turn of the 1970s, the Institute of Gerontology in Kyiv had established itself as a leading centre of international gerontological research and geriatric medical practice. The Institute hosted training courses not only for Soviet doctors, but for those from ‘near’ and ‘far’ abroad; it conducted medical and biological research that was known and cited in Western journals. In particular, the biological gerontologist Venyamin Frol’kis and the demographer Nina Sachuk were frequent contributors to foreign journals. As Alex Comfort, a leading British gerontologist and the founding editor of *Experimental Gerontology*, wrote to Chebotaryov in 1968, ‘We would be delighted to have more Soviet papers … nothing would please us more than to print at least one good Soviet paper in each of our annual issues …. certainly we in England are determined to maintain the closest and friendliest contacts with your research’.^34^ Comfort’s estimation was echoed by Akhmeteli’s successor as head of the WHO European office’s Division of Chronic Diseases, Z. Pisha, who found the Institute a ‘wonderful place’ that left a ‘serious impression’ on the visitor.^35^ Anderson (2007: 128) was impressed by the funding and development of gerontology in Kyiv, as well as by Chebotaryov, whom he ‘much admired’. Shuman continuously promoted the Institute in Kyiv, stating he was ‘very impressed by’ its work, especially in terms of its ‘unique programme combining gerontology and geriatrics’.^36^ Chebotaryov also found allies in the IAG, and by the late 1960s, he was able to arrange that the 9^th^ Annual Congress of the Institute, planned for 1972, was held in Kyiv.

## The ‘abroad’ looks to Soviet Kyiv (1972–1986)

Chebotaryov had spent years working to organise an IAG congress in Kyiv. He made his first attempt in 1963, writing to the Academy of Medical Sciences for support – but his appeal went unanswered.^37^ Since working through official Soviet channels was less than successful, Chebotaryov made something of a ‘gamble’ (*avantiura*): he first arranged matters with the IAG and only thereafter informed the authorities in Moscow. In Vienna for the 7^th^ Congress of the IAG, Chebotaryov was involved in discussions about where to hold the 9^th^ Congress in 1972. Seizing the opportunity and ‘understanding that at this moment much was at stake, in fact whether or not our Institute in Kyiv would gain international status’, Chebotaryov made the formal suggestion that the 9^th^ Congress were held in Kyiv (Chebotarev, 2008: 96). Although initially sceptical, the President of the IAG, the Austrian gerontologist Walter Doberauer, promised to consider the possibility, and made a trip to Kyiv in early 1967 to investigate. Pleasantly surprised by the scale of work being done in Kyiv and the space available to hold the congress, Doberauer and the IAG gave their approval. Only after this, armed with official documents from the IAG, did Chebotaryov ‘write directly to the Minister of Healthcare, Boris Vasil’evich Petrovsky, letting him know that, you know, there is the entirely real idea of holding the Congress in Ukraine, especially considering that Kyiv has become one of the world’s scientific centres of gerontological study’ (Chebotarev, 2008: 97). Faced with what seemed an already done deal, both the Council of Ministers of the USSR and the Central Committee of the Communist Party of the Soviet Union (CPSU) gave their formal approval in October 1967.^38^ The IAG then made its own announcement of the planned congress in early 1968, informing the world of Kyiv’s place as a ‘centre of gerontological study’.^39^

The 9^th^ Congress of the IAG was held in Kyiv in July 1972. More than 2,500 gerontologists, geriatricians, and doctors took part, including approximately 1,500 from elsewhere around the world. This included around 500 Americans, 600 Western Europeans, 50 Australians, and numerous participants from Latin America and Asia.^40^ The leading lights of international gerontology – Alex Comfort and William Ferguson Anderson from the UK; Nathan Shock, Leonid Hayflick, and William Cowdry from the US; Walter Doberaur, R. J. van Zonnenveld, Fritz Verzar, and others from Europe – were all in attendance. Nearly any Soviet scientist who worked in a field related – even tangentially – to gerontology made an effort to attend, including those who were forced to use their vacation days to do so.^41^ Overall, most estimations of the proceedings were positive, and both the organisation and the quality of papers presented were evaluated as befitting expectations – although there were some complaints made about the lack of a translation service and the inability for many Soviet participants to communicate in English (Shock and Baker, 1988: 165). There was also a moment of hubbub when Zhores Medvedev, a famous dissident and gerontologist who had been invited to the Congress by the IAG but uninvited by Chebotaryov and the Soviet organisers, tried to attend anyway and was forcibly removed by KGB agents (Chebotarev, 2008: 116–17; Medvedev, n.d.: Ch. 16; Shock and Baker, 1988: 180–5). Notwithstanding the collective anger expressed over his treatment by Shock and other Western gerontologists, this event seemed to have few long-term repercussions for the Institute in Kyiv or its standing in world gerontology.

On the contrary, Chebotaryov was accurate when he later wrote that ‘The congress was that final push, which put our Institute on the international map’ (Chebotarev, 2008: 115). As the host of the 1972 Congress, Chebotaryov became the president of the IAG for the next three years (1972–1975), a position that allowed him to both increase the Institute’s international status and promote its particular agenda in the field of gerontology and geriatrics. As the 1963 WHO Seminar in Kyiv had outlined, and as numerous Soviet publications over the subsequent decades highlighted, this vision of gerontology was of a science built on an assumption of interrelatedness between older persons and their surroundings: home, work, social order, and environment. As this article has argued, the Soviet vision of gerontology was thus a dialectical theory of ageing that also suggested practical relevance to older persons. It was not a science restricted to the laboratory or to the clinic, but one that could actually *improve* human lives by considering the ways in which processes of ageing were connected to daily life. As Chebotaryov summarised then, the idea was to help ‘older persons age normally’, which meant living healthily and satisfactorily, approaching ageing not as a disease to be treated or a biological barrier to be fought against, but instead a period of life to be understood, defined, and maximised in terms of its benefit for older persons. Organisationally, this entailed greater emphasis on research into the social lives of and the therapeutic practices relevant to older persons; it also suggested an increasingly close relationship with intergovernmental organisations, which were directly involved in the promotion of such activities.

Chebotaryov used his position as the head of the IAG to solidify these relationships. Building on previous consultations with Shuman at the UN Unit on Ageing, a formal relationship was established between the Institute of Gerontology in Kyiv and the Unit in 1972. The IAG also formed an UN–IAG Liaison Committee under Chebotaryov’s oversight, and over the next few years also increased its status vis-à-vis the UN, ultimately registering as a ‘category I’ NGO, that is, an NGO with full consultative and coordinative rights with UN bodies (Shock and Baker, 1988: 234). In 1973, moreover, the WHO formed its first Committee on Planning and Organization of Geriatric Services, which was meant as a coordinative body for NGOs working on ageing. The IAG was included, with Chebotaryov as the chairman of its delegation, where he was joined by his old collaborators, Dr Anderson (Scotland) and Dr Zonneveld (the Netherlands) (Chebotarev, 2008: 147; Shock and Baker, 1988: 178).

Even after the end of Chebotaryov’s IAG presidency in 1975, the Institute in Kyiv remained closely engaged with international organisations. The WHO’s Committee on Planning and Organization of Geriatric Services met frequently, as did coordinative bodies made up of the directors of leading gerontological centres, whose meetings were organised by the WHO in Copenhagen in 1976, in Tokyo in 1978, in Weimar, GDR, in 1980, and in Bethesda, USA, in 1977 and 1981 (Chebotarev, 2008: 147). In 1974, the UN Unit on Ageing asked the Institute of Gerontology in Kyiv to lead an interregional UN Seminar on Ageing and Practices of Social Support for Older Persons for representatives of developing nations. This seminar was finally held in May 1979 and brought together representatives of 20 countries in Kyiv. The seminar was jointly led by Vladislav Bezrukov from the Institute of Gerontology and Enver Ergun, then the deputy director of the UN Centre for Social Development and Humanitarian Affairs in Vienna; participants were led through a programme that emphasised the Soviet approach to ageing and the mix of social, economic, and medical support that this implied (Chebotarev, 2008: 149).^42^ The late 1970s also saw the development of joint UN–WHO programmes related to the provision of medical and social services to older persons and the prevention of premature ageing, ideas that reflected earlier concepts promoted by the Institute of Gerontology and where Chebotaryov was closely involved in the discussions held in Vienna and New York City. He also arranged at the same time for the creation of ‘research centres’ in Kyiv under the auspices of the WHO related to ‘older persons in the family and society’, ‘medicinal treatment for older patients’, and ‘preventing premature ageing (biological and social aspects)’ (Chebotarev, 2008: 148–9). This tied the research work being done at the Institute to the international agenda of the WHO (and, not incidentally, to the funding available through this organisation).

By the early 1980s, the Institute of Gerontology was solidly within the leading circles of international geriatrics and gerontology. Following the founding of the National Institute of Ageing (NIA) as part of the US National Institutes of Health, there began a mutually amicable and frequent exchange of visitors between the institutes, with the founding Director Dr Robert Butler visiting Kyiv twice in 1978 and 1980 (Achenbaum, 2013: 139; Chebotarev, 2008: 81).^43^ In addition to Chebotaryov’s clearly prominent international role, both Nina Sachuk and Vladislav Bezrukov had also become formal consultants to the WHO. Venyamin Frol’kis and O. V. Korkusho, amongst the Institute’s most prominent scientists, were on the editorial boards of international (Western) academic journals.^44^ The Institute’s research continued to be published abroad and its ideas were cited and spreading. In the particular field of social support for older persons and advocacy for their needs, moreover, the impact was undeniable. After 1973, as Nathan Shock later groused,

the IAG devoted more attention to the political and social issues associated with ageing than had been true previously … [there was] a frank call for the support of political actions to improve the lot of elderly people in all nations of the world. The IAG became an advocate for specific programs for the aged based on political rather than scientific considerations. (Shock and Baker, 1988: 178, 223)

For those like Shock, this may have represented an unfortunate deviation from the origins of the IAG and gerontology as a laboratory science, but it was clearly in line with and partially the result of the Soviet approach to finding ways to allow ‘older persons to age normally’ and to use social, medical, and other services to avoid premature ageing.

This impact was also clear during the UN World Assembly on Ageing, held in Vienna in July–August 1982. Exactly a decade in the making, the idea for a world assembly had initially been floated in discussions between Chebotaryov and US Senator Frank Church during the 1972 IAG Congress in Kyiv (Chebotarev, 2008: 150; Shock and Baker, 1988: 219).^45^ This led to a flurry of meetings over the following years, with the Institute of Gerontology in Kyiv hosting some, and Chebotaryov visiting others; the IAG also continued to lobby for the Assembly at the UN.^46^ In December 1978, the UN General Assembly voted to hold a World Assembly on Ageing in 1982 (UN, 1978). When held, this was an enormous and formal affair, providing the opportunity for representatives of 124 countries to express their views on the challenges caused by the ‘demographic revolution’ and the unabating increase in the number of older persons worldwide (UN, 1982a: 6–7). There was some sense, however, that some states may have been more invested than others. With the American General Secretary of the International Federation of Aging (IFA), William Kerrigan, appointed Secretary-General of the World Assembly, and with most of the funding coming from the US, the 1982 World Assembly has in retrospect been largely seen as an American and Western project.^47^ The influence of alternative visions on ageing, it has been suggested, was hardly visible.^48^

This, however, is inaccurate. While the American impact on the World Assembly may have been more obvious, under the surface, the actual organisation and content of the proceedings and their output were the product of a much more complex mix of influences, including, most notably, Kyiv’s. One of Kerrigan’s deputies in charge of the World Assembly’s organisation was Vladislav Bezrukov, by then, Chebotaryov’s deputy director in Kyiv.^49^ Chebotaryov himself, having decided to avoid a more visible (and thus politicised) role at the Assembly, headed up the Ukrainian delegation – and also served as the deputy chairman of the ‘Main Committee’ or working group of the Assembly (Chebotarev, 2008: 153; UN, 1982a: 37). It was this ‘Main Committee’ that summarised the discussions and findings of the Assembly into its published report and ‘International Plan of Action on Aging’, a document that strongly reflected decades of Kyiv’s research and advocacy on matters of aging. Recommendations in this Plan of Action emphasised nutrition, exercise, and therapeutic treatments, much as Kyiv had promoted over the decades (UN, 1982a: 61). Standardised categories of old age, starting from 60 and distinguishing between those persons simply ‘old’ and those ‘old old’, were employed, equally reflecting the earlier categories introduced in Kyiv in 1963. Strikingly, moreover, the Plan of Action emphasised that ‘Particular attention could be given to co-ordinating preventive efforts in order to combat the detrimental effects of *premature aging*’ (UN, 1982a: 60; emphasis added). This aligned with the broader underlying philosophy of the Plan of Action (and of the World Assembly) that ‘Quality of life [is] no less important than longevity, and that the ageing should, therefore, as far as possible, be enabled to enjoy in their own families and communities a life of fulfilment, health, security, and contentment’ (UN, 1982a: 47; 1982b). In this reading, ageing was neither a disease to be treated nor an undesirable stage of life to be put off through the biomedical prolongation of youth – as the Western biomedical model of ageing, dominant in the 1980s and outlined in this article’s introduction, would have suggested – it was instead a physiological process to be made as normal as possible. In line with the Soviet theory of dialectical ageing, moreover, this physiological process was inherently connected to ‘families and communities’ as well as work environments and older person’s surroundings, all of which were key points of emphasis in the World Assembly’s final documents. As Tarek Shuman, the head of the UN Unit on Ageing and background organiser of the World Assembly, later remarked, it was this Soviet concept of ‘ageing as a process … [that] was reflected in the Vienna Plan of Action’, and made clear Chebotaryov’s and Kyiv’s central role.^50^

For a few years after the 1982 World Congress, the Institute of Gerontology in Kyiv retained its position at the centre of international gerontology. In 1980, the UN Unit on Aging was joined by a formal WHO Programme on Health of the Elderly (IRP/HEE), which began to coordinate efforts across different national gerontological centres (Mikhailova, 2003: 32). With Chebotaryov, Bezrukov, and Sachuk all working as WHO consultants, they were initially closely involved with the WHO programme, and in the early 1980s, Kyiv was considered to be the WHO’s main ‘Collaborating Centre’ for research on ageing in Europe.^51^ The Institute for Gerontology in Kyiv was also listed by the WHO as one of three ‘giants’ of ageing-related research worldwide (along with Bethesda and Tokyo).^52^ This ongoing influence on international gerontology could be seen through the continued participation of Kyiv’s scientists at international meetings (for example, in Stockholm) and the holding of a second Interregional UN Seminar on Aging in Kyiv in 1985 (Chebotarev, 2008: 154–5). When Tarek Shuman retired from his position as head of the UN Unit on Aging in 1986, moreover, he recommended and was replaced by Vladislav Bezrukov.^53^ And the underlying philosophy embedded in WHO and UN programmes on ageing continued to reflect Soviet ideas of ‘normal ageing’ as a process dialectically integrated into the life course, as a 1986 summary of the WHO Programme on Health of the Elderly suggested:

The major re-orientation of the current programme is signalled by its change of name to ‘Health of the Elderly’. This change aims to diminish any preoccupation with medical care of the disease conditions of old age and to emphasize rather health promotion, disease prevention and disability postponement throughout the life course.^54^

## Fading prestige and influence after 1986

At the same time, however, the role of Kyiv as a ‘giant’ of gerontological research worldwide was already beginning to fade. This was due to both personal and geopolitical reasons: relationships developed by Chebotaryov started to diminish in importance at the same time that the USSR’s own fortunes, economic and political alike, began to wane. With no ally in the WHO similar to Akhmeteli, Bezrukov found it difficult to develop a close working relationship between the UN Unit on Aging and the WHO Programme on Health of the Elderly. Correspondence between the two agencies, in fact, tended to demonstrate a lack of communication.^55^ The WHO, moreover, began to increasingly organise expert committee meetings and seminars on ageing without inviting experts from either the UN or Kyiv, instead replacing the latter with cardiologists and less-specialised experts from Prague (perhaps not incidentally, the Head of the WHO Programme on Health of the Elderly in the 1980s, Dr Hana Hermanova, was from the Czech Republic).^56^ When experts from Kyiv were invited, it could seem to be as an afterthought. In November 1987, for example, the WHO organised a WHO Expert Committee on Health of the Elderly meeting in Geneva, to which it invited Anatoly Tokar’, then the acting director of the Institute of Gerontology in Kyiv – but only because a certain Dr Linos Shcallis of Cyprus had been unable to attend.^57^ This reflected the broader approach to the Institute in Kyiv on the part of the WHO. The final ‘Special Programme for Research on Aging’ developed by the WHO in late 1987 restricted its partnerships to the NIA in Washington and a clinic in Prague, essentially cutting out the Institute in Kyiv, notwithstanding Chebotaryov’s efforts to help establish WHO programmes in the field of ageing in the 1960s and 1970s.^58^ This situation was exacerbated in early 1988 when Chebotaryov retired from his position as the head of the Institute in Kyiv and Bezrukov was recalled to take over the directorship, leaving neither Soviet citizens nor close colleagues in either of the major international organisations involved in ageing and gerontology.^59^

This lack of personal connection overlapped with changes in both the basic approach of international organisations to state institutions and the institutional capacity of the Soviet Union. The 1980s was a time of broad ‘NGO-isation’ of development aid, international coordination, and ‘capacity-building’ in international organisations. The UN and other organisations encouraged their agencies to work more closely with NGOs instead of state institutions, part of a larger emphasis on the former’s supposed relative efficiency and sustainability (Davies, 2013: 141–54). This was equally true in the field of ageing, gerontology, and geriatric medicine. The 1970s and 1980s had seen significant growth in the number of NGOs working in the field of aging, and by the mid-1980s, coordination with these organisations (instead of state institutions like the Institute of Gerontology in Kyiv) began to dominate the activities of the WHO and the UN. As Dr David Macfadyen of the WHO Programme on the Health of the Elderly remarked in 1985, ‘We have been especially fortunate in the Health of the Elderly programme in finding helpers, notably in nongovernmental organizations’. It was with these NGOs, Macfadyen argued, that the WHO was now pursuing its work in relation to older persons.^60^ This left increasingly little space for institutions like the Institute in Kyiv – and in fact put socialist states, where NGOs were very poorly developed, at a distinct disadvantage (see, broadly, Sievers, 2003).

This disadvantage was made worse by the financial and economic chaos gripping the USSR throughout the latter years of the 1980s. Financial support for gerontology and ageing had been at best marginal throughout Soviet history, and there had always been issues coordinating payments with the UN and WHO. By the late 1980s, moreover, the Soviet Union’s federal budget was in deficit and republican governments found themselves hardly able to tread water. There was clearly no excess capacity to support international collaboration, a situation Bezrukov faced when discussing with the WHO and UN the founding of the joint International Institute on Aging (INIA) in 1987. Representing a culmination of efforts began at the 1982 World Assembly and the WHO research programmes of the 1980s, the INIA was meant to act as the central hub for international research on gerontology and advocacy on behalf of older persons. Initially, discussions had considered basing the INIA in Kyiv, given its status as an internationally recognised centre in this field. When it became clear that the Soviet government was not in a position to provide funding, however, the UN quickly shifted to another candidate, Malta, which was not famous as a centre of gerontological research but whose politicians had often voiced support for ageing-related issues at the UN – and whose state was willing to provide financial backing.^61^ As a result, the INIA was opened in Malta in April 1988; its subsequent meetings, seminars, and expert committees included no representatives from Kyiv.^62^

## Conclusion: Soviet gerontology *sine qua non*

Since the collapse of the USSR, historical memory has tended to forget Kyiv’s role in the development of international gerontology and efforts taken by the UN, WHO, and other international organisations to improve the lives of older persons worldwide. In part, this is a product of the authors writing the history of international science, and gerontology in particular: the Maltese, as the founders and directors of the INIA, emphasise the importance of Malta, much as they have done since 1989; Americans suggest the same for the US as the real ‘driver’ of events such as the 1982 World Assembly. Histories of international gerontology, written largely in the US and Europe, focus on scientists, doctors, and organisations based in the ‘Western’ world. Because the USSR collapsed, in other words, its influence has become obscured, there are no longer individuals or advocates working in the field of gerontology interested in promoting its legacy. In addition, moreover, since the collapse of the USSR in 1991, there has been a broader delegitimisation of socialist ideas and the idea of ‘socialist development’ writ large. Considered to have been representations of a political dead-end, as Maria Cristina Galmarini (2024: 2) has written, there has been a widespread ‘erasure of socialist actors’ contributions to the articulation of social progress ideas’. Yet, as Galmarini evocatively shows in the case of blind advocacy groups – and as others have recently demonstrated in relation to polio vaccines (Vargha, 2018), deaf activism (Shaw, 2021), and conceptual frameworks for disability (Baar, 2021) – in the medical sciences, it was frequently the international interplay between socialist and Western actors and networks that ‘was a driver of transformation’ (Galmarini, 2024: 3) in capitalist states. Complicating the binary vision of Western scientific development challenged only by developing states, this new literature has built on an earlier – if overlooked – body of scholarship on the importance and influence of Soviet and socialist science across many disciplines (e.g. Graham, 1985, 1987; Kojevnikov, 2004).

The story of the Institute of Gerontology in Kyiv and its influence on international agencies and the worldwide approach to ageing dovetails with this more complex and intertwined history. As this article has shown, Soviet gerontology developed over decades both in close collaboration with international scientific circles and in a theoretically specific fashion. Its emphasis on the dialectical interaction between an older person and her or his physical, social, and work environments provided the foundation for an internal programme of research and intervention, carried out in Kyiv and elsewhere. As Susan Grant (2022: 208–9) has written, this programme was both coherent and unique: ‘The integrated and in-depth nature of the Kyiv-based Institute of Gerontology’s research on the role of exercise, health and ageing arguably set the Soviet Union apart’. This article has outlined how this vision of ageing as a dialectical but inherently *normal* aspect of life was developed in Kyiv – and how the influence of the Soviet theory of ageing was then passed to international scientists, researchers, and development workers. As this article has shown, moreover, the Soviet Union and its dialectical ideas about ageing were initially and extensively present during the establishment of international programmes on ageing at the UN and WHO, providing an alternative to the biomedical determinism in vogue in the 1970s and 1980s in the US and elsewhere.

These ideas, including an emphasis on the lived experience of ageing – nutrition, exercise, labour, healthcare, etc. – filtered into the work of international gerontological organisations, the UN Programme on Ageing, and the WHO’s specialised programmes. They were present in the recommendations of the 1982 World Assembly on Ageing, and, as Susan Grant (2022) has also emphasised, reflected in the 2007 Assembly in Madrid. Today, many studies of the ‘demographic revolution’ that continues to increase the place and importance of older persons globally have embraced a vision of geriatric medicine and gerontology that rejects biomedical determinism and the idea of ageing as an inevitable decline. Instead, there has been a renewed focus in the past few decades on the importance of social order, and of the interplay between older persons and their environments as one of the central factors determining medical and physiological outcomes in older age (e.g. Armstrong, 2019; Buettner, 2008; Finch, 2007). The idea of old age as a normal part of the ageing process is also emphasised – even something, as Louise Aronson (2019) has eloquently argued, to be *embraced* as part of the entire life course. These works do not connect their ideas of interaction between older persons and their environment to the Soviet Union or previous conceptions of ageing. With the benefit of hindsight, however, it becomes clear that these ideas are not as new as they are being presented: they follow a clear trajectory from Kyiv through Copenhagen, New York, and Vienna to the rest of the increasingly ageing world today.

## Notes

1. Relatively little has been written about the Soviet Union’s relationship with the UN, but scholarship (e.g. Brinkley, 1970; Gaiduk, 2013) tends to agree on a sceptical approach.

2. Calculated from UN data (1982a: 50). The UN’s figures from 1982 turned out to be relatively exact, predicting with reasonable accuracy both the world population (approximately 8 billion) and the total number of older persons (over 1 billion) living 40 years later.

3. Life expectancy in the Netherlands in 1982 was 76; in the US, it was 75; in France, it was also 75; in (Soviet) Russia, the figure was 68, while in (Soviet) Ukraine it was 70. As per data held in the Human Mortality Database (https://www.mortality.org).

4. The ‘anti-reticular cytotoxic serum’ (*antiretikuliarnaia tsitotoksicheskaia syvorotka*) was derived from the blood plasma of animals (often goats or rabbits), which had been treated with antibodies taken from the spleens of other young animals. It was found to improve the healing of wounds when given in small dosages, and later promoted as an ‘anti-ageing’ treatment, although with little evidence to support this application. For a description of its uses and results, see Bogomolets (1940: 124–35).

5. On the work of Bogomolets and Nagornyi in the 1920s–1940s, see Bezrukov and Duplenko (2023: 20–1).

6. Also see Dubinin’s May 1955 criticism of Lysenko, sent to Viacheslav Molotov and the Central Committee of the Communist Party of the Soviet Union (CPSU), as held in the Russian Government Archive of Contemporary History (RGANI) f. 5, op. 17, d. 516, ll: 3–7. Loren Graham (1987: 140–2) has also argued that Lysenko’s influence was significantly reduced after 1956.

7. Bogomolets died in 1946, while Nagornyi passed away in 1953.

8. The K. Parkhon Memorial Institute was famous at the time for the work of its director Ana Aslan and her development of ‘lidocaine treatments’ for ailments common in older age, which were much discussed (and later largely dismissed) internationally.

9. On the trip to Romania, see Otchet sovetskoi meditsinskoi delegatsii o poezdke v Rumynskuiu narodnuiu respubliku, g. Bukharest [Report on the Soviet medical delegation on its trip to the Romanian People's Republic, Bucharest], State Archive of the Russian Federation (GARF) f. 8009, op. 34, d. 370, l: 1; for the trip to Italy, see Otchet delegatsii o poezdke na mezhdunardonyi kongress gerontologov v Italiiu [Report of the delegation on its trip to the international congress of gerontologists in Italy], GARF f. 8009, op. 34, d. 319, l: 2.

10. Otchet delegatsii o poezdke na mezhdunardonyi kongress gerontologov v Italiiu [Report of the delegation on its trip to the international congress of gerontologists in Italy], GARF f. 8009, op. 34, d. 319, ll: 12–13.

11. Sostav nauchno-metodicheskogo komiteta po gerontologii i geriatrii pri UMS Ministerstva zdravookhraneniia SSSR i AMN SSSR na 1957-1958 gg. / I polugodie/ [Composition of the scientific-medical committee for gerontology and geriatrics of the UMS, Ministry of Healthcare of the USSR and the Academy of Medical Sciences of the USSR, 1957-1958 / first half year/], GARF f. 8009, op. 2, d. 2347, l: 6.

12. Plan raboty nauchno-metodicheskogo komiteta po gerontologii i geriatrii pri UMS Ministerstva zdravookhraneniia SSSR i AMN SSSR na 1957–1958 gg. / I polugodie/ [Work plan for the scientific-medical committee for gerontology and geriatrics of the UMS, Ministry of Healthcare of the USSR and the Academy of Medical Sciences of the USSR, 1957-1958 / first half year/], GARF f. 8009, op. 2, d. 2347, l: 8.

13. For the February 1957 agreement with the Ukrainian Academy of Medical Sciences (AMN), see Spravka o vypolnenii resheniia Kollegii Ministerstva zdravookhraneniia Soiuza SSR ot 16 maia 1957 goda ‘O meropriiatiiakh po probleme stareniia i dolgoletiia v SSSR’ [Report on the fulfilment of the resolution made by the College of the Ministry of Healthcare of the USSR on 16 May 1957, ‘On activities related to the problem of ageing and longevity in the USSR’], dated 12 March 1958, GARF f. 8009, op. 2, d. 2347, l: 10. For the arguments in favour of other cities, see GARF f. 8009, op. 2, d. 2347, ll: 17–19, 28–32.

14. As characterised by his frequent collaborator, the Scottish geriatrician Sir William Ferguson Anderson. See Report to the WHO by Sir William Ferguson Anderson on the ‘Course on the Medical and Social Aspects of the Care of the Elderly, EURO 2591’, Central State Archive of Supreme Bodies of Power and Government of Ukraine (TsDAVO) f. 4783, op. 1, d. 220, l: 78. For his early biography, see Chebotarev (2008: 5).

15. The Ministry of Health’s Fourth Main Directorate was responsible for treating party leaders; it involved close contact with security services, including the KGB. See Scarborough (2022: 1250).

16. The reasons for the change in leadership at the Institute remain ambiguous. Chebotaryov argued that Gorev decided that he preferred experimental work and chose to leave the administrative post. Vladislav Bezrukov, however, who joined the Institute around this time and later became its third director, suggested that Gorev was ‘promoted’ to a position in the Academy of Medical Sciences in Moscow, but found life in the Soviet capital too hectic, and thus returned to Kyiv but to a lower position at the Institute. For this version of events, see author's interview with Vladislav Bezrukov, Kyiv, September 2019.

17. ‘Mikhail Andreevich AKHMETELI’. WHO Employment file. WHO Archive, Geneva.

18. Letter from Dr P. van de Kalsaid, Director of the WHO Regional Office for Europe to D. F. Chebotarev, dated 23 January 1963. TsDAVO, f. 4783, op. 1, d. 106, l: 1; Chebotarev (2008: 93–5).

19. ‘Prilozhenie: Spisok uchastnikov’ [Appendix: List of participants], TsDAVO f. 4783, op. 1, d. 103, ll: 18–20.

20. Ivan Tarkhnishvili (Tarkhanov), a Georgian physician and anatomist working in St Petersburg at the end of the 19^th^ century, published a series of articles in *Vestnik Evropy* in 1891, in which he outlined his thoughts on biological ageing and, in part, ‘premature ageing’. These ideas were later expanded by Bogomolets, Chebotaryov, and others. See Pitskhelauri (1968: 24–6).

21. For the content of the seminar, see Okhrana zdorov’ia pozhilykh i starykh liudei i preduprezhdeniie prezhdevremennogo stareniia. Otchet o Seminare, sozvannom Vsemirnoi organizatsiei zdravookhraneniia. Kyiv, 14–22 maia 1963 goda [Protection of the health of older and elderly people and the prevention of premature ageing. Report on the Seminar, called by the World Health Organization, Kyiv, 14-22 May 1963], TsDAVO f. 4783, op. 1, d. 1–3, ll: 1–17; Otchet o provedenii Evropeiskogo seminara po okhrane zdorov’ia pozhilykh i starykh liudei i preduprezhdenie prezhdevremennogo stareniia [Report on the activities of the European seminar on the protection of the health of older and elderly people and the prevention of premature ageing], TsDAVO f. 4783, op. 1, d. 103, ll: 20–33. Age categories on pages 6, 23. Also see D. F. Chebotarev, ‘Development of Gerontology and Geriatrics in the USSR’, report to the Seminar on the Health Protection of the Elderly and the Aged and the Prevention of Premature Ageing, Kyiv, 14–22 May 1963. WHO Archive, EURO-245/15, 9 May 1963; Chebotarev, 2008: 93.

22. See the report of the Soviet delegation to the VI World Congress of the International Association for Gerontology, held in Copenhagen, Denmark, in August 1963. TsDAVO, 4783-1-109-16; 4783-1-109-10.

23. TsDAVO, 4783-1-103-23.

24. See, for example, the letter from Robert Proper, M.D. of Albuquerque, US, sent to D. F. Chebotaryov on 06.09.1963, in which such a visit from Shock to Kyiv was discussed. TsDAVO, f. 4783, op. 1, d. 105, l: 146.

25. TsDAVO f. 4783, op. 1, d. 146, l: 53.

26. On Akhmeteli’s work with the Ministry of Healthcare in 1965, see TsDAVO f. 4783, op. 1, d. 146, l. 3; Mikhail Andreevich AKHMETELI. WHO Employment file. WHO Archive, Geneva.

27. TsDAVO f. 4783, op. 1, d. 146, l: 14.

28. On the content of the 1965 course, see Programma kursov po meditsinskim i sotsial’nym voprosam gerontologii [Programme of courses covering medical and social aspects of gerontology], TsDAVO f. 4783, op. 1, d. 146, ll: 6–9; on the number of total hours and dates, see TsDAVO f. 4783, op. 1, d. 146, l: 11.

29. On Anderson’s participation in the 1968 courses, see his letter to Chebotaryov, sent on 12 January 1968. TsDAVO f. 4783, op. 1, d. 219, l: 6; on Zetterqvist’s, TsDAVO f. 4783, op. 1, d. 219, l: 24.

30. Verzar was a Hungarian-born Swiss gerontologist who had done much to develop gerontology as a discipline in Central Europe (see Robert, 2006; Shock and Baker, 1988: 109–14). On his and Shock’s visit to Kyiv in 1968, see Letter from F. Verzar to D. F. Chebotarev, 04.03.1968, TsDAVO f. 4783, op. 1, d. 218, l: 46; Letter from D. F. Chebotarev to N. Shock, 3 April 1968, TsDAVO f. 4783, op. 1, d. 218, l: 56. The Swedish gerontologists Irma and Per-Olaf Astrand also attended; TsDAVO f. 4783, op. 1, d. 218, l: 116.

31. Chebotaryov became a formal consultant to the WHO in 1962 and remained in this role until 1983 (see Chebotarev, 2008: 95).For his part, while still a young researcher at the Institute in Kyiv, Bezrukov was frequently recruited to accompany visitors flying through Moscow to Kyiv, including Ferguson; see the author's interview with Vladislav Bezrukov, Kyiv, September 2019; Anderson (2007: 128).

32. TsDAVO f. 4783, op. 1, d. 218, l: 21; personal letter (email) sent from Tarek Shuman to the author, 1 August 2019.

33. A formal relationship between the UN and the Institute of Gerontology in Kyiv was established in 1972 (see Chebotarev, 2008: 151).

34. Letter from A. Comfort to Professor Chebotarev, 1 January 1968, TsDAVO f. 4783, op 1, d. 218, l: 7. On Comfort’s work in gerontology more broadly, see Moreira (2019).

35. Letter from Z. Pisha, WHO, to D. F. Chebotarev, 22 January 1965, TsDAVO f. 4783, op. 1, d. 146, l: 15.

36. Personal letters (emails) sent from Tarek Shuman to the author, 1 August and 6 September 2019.

37. Pis’mo D. F. Chebotareva Rukovoditel’iu otdela mezhdunarodhnykh nauchnykh sviazei AMN SSSR professoru S. A. Sarkisovu [Letter from D.F. Chebotaryov to the Director of the Division of International Scientific Connections of the Academy of Medical Sciences of the USSR, professor S.A. Sarkisov], 22 May 1963, TsDAVO f. 4783, op. 1, d. 105, l: 76.

38. See the Postanovlenie Soveta Ministrov SSSR ot 4 oktiabria 1967 [Act of the Council of Ministers of the USSR, 4 October 1967], as referenced in Stenogramma zasedaniia Komissii po podgotovke provedeniia IX Mezhdunarodnogo Kongressa gerontologov [Transcript of the session of the Commission for the preparation of the Ninth International Congress of gerontologists], 18 October 1971. TsDAVO f. 2, op. 13, d. 5554, l: 114.

39. Letter from D. F. Chebotarev to N. Shock, 3 April 1968, TsDAVO f. 4783, op. 1, d. 218, l: 56

40. Numbers of registered participants at the Congress surprisingly vary by source, but most reports place the total between 2,500 and 2,600, with approximately 1,000–1,100 Soviet participants and 1,500–1,600 foreign citizens, including somewhere around 1,200 ‘Westerners’. For divergent figures, see Chebotarev (2008: 103–4); Shock and Baker (1988: 154–6); Otchet Predsedatelia Natsional’nogo orgkomiteta, Zamestitelia Predsedatelia Soveta Ministrov USSR, P. Tron’ko v Sovet ministrov Soiuza SSR tovarishchu Kosyginu A.N. ‘O provedenii IX Mezhdunarodnogo kongressa gerontologov’ 4.8.1972 [Report of the Chairman of the National organizational committee, Vice-Chairman of the Council of Ministers of the Ukrainian SSR, P. Tron'ko to the Council of Ministers of the USSR, comrade A.N. Kosygin 'On the activities of the Ninth International Congress of gerontologists', 04 August 1972], TsDAVO f. 2, op. 13, d. 6716, l: 156.

41. As reported, for example, by both Zhores Medvedev (n.d.: Ch. 16) and the theoretical biologist Aleksei Olovnikov (in Hall, 2003: 46–7).

42. Ergun was a Turkish national who was later assassinated in Vienna in 1984 by an Armenian radical. See Foulds (1984).

43. Also see the author's interview with Vladislav Bezrukov, Kyiv, September 2019.

44. ‘Ob itogakh deiatel’nosti i zadachakh nauchno-meditsinskogo obshchestva regontologov i geriatrov’ (otchetnyi doklad predsedatelia Pravleniia Vsesoiuznogo obshchestva gerontologov i geriatrov, akademika AMN SSSR D.F. Chebotareva IV Vsesoiuznomu s’’ezdu gerontologov i geriatrov, Kishinev, 1982) [‘On the results of the activities and responsibilities of the scientific-medical society of gerontologists and geriatricians' (Summary report of the Chariman of the Administration of the All-Union society of gerontologists and geriatricians of the Academy of Medical Sciences of the USSR, D.F. Chebotaryov to the Fourth All-Union congress of gerontologists and geriatricians, Chisinau, 1982], 17 September 1982, GARF 8009, 55, 78, 42.

45. Also see personal correspondence (email) to the author by Tarek Shuman, 6 September 2019.

46. On IAG lobbying, see Shock and Baker (1988: 219); for planning meetings involving Chebotaryov, see Chebotarev (2008: 150–1). One of the few authors to have written about the planning for the World Assembly, Kavita Sivaramakrishnan (2018: 274), has also noted these meetings, although she erroneously elevates the role of Ethel Shanas, an American appointed by Chebotaryov to chair of the UN–AIG Liaison Committee, over Chebotaryov himself and generally disregards the Soviet influence.

47. Kerrigan was appointed as the Secretary-General of the World Assembly in June 1981; see UN (1982a: 3). Funding for the 1982 World Assembly was not provided for in the UN’s annual budget but was instead covered by a ‘voluntary fund for the World Assembly on Aging’, to which the US was the largest donor. On this point, see UN (1980); author's interview with Vladislav Bezrukov, Kyiv, September 2019; personal correspondence (email) from Tarek Shuman to the author, 1 August 2019.

48. This dominant position of the ‘American’ views on ageing may be interpreted positively, as was often the case in the 1980s, or negatively, as is now more frequent. But the possibility of Eastern European or socialist influences is largely dismissed. For the positive interpretation of the ‘American’ agenda at the 1982 World Assembly, see Kerschner (1988); for a later critical view, see Sivaramakrishnan (2018: 154–60).

49. Personal correspondence (email) from Tarek Shuman to the author, 1 August 2019.

50. Personal communication (email) from Tarek Shuman to the author, 6 September 2019.

51. ‘WHO Collaborating Centres for Health of the Elderly’, WHO Activity Report, 9 September 1988, no 9008E / 7.88, WHO Archive EURO-101 / IRP-HEE 100.

52. ACMR Subcommittee on Research on Aging, ‘Proposed International Research Programme on Aging: Detailed Justification’, 21 March 1986, no 4946R, p. 5, WHO Archive, EURO-071, IRP/HEE/114.5.

53. ‘Note for the File’, 3 February 1986, WHO Archive EURO-086, IRP/HEE 116.03.

54. ‘Address by the World Health Organization to the NGO/UN Liaison Committee on Aging, Vienna on the Programme on Health of the Elderly, 1984–1985’, WHO Archive EURO-086, IRP/HEE 116.03, address p. 1.

55. ‘Note for the File’, 3 February 1986, WHO Archive EURO-086, IRP/HEE 116.03.

56. See, for example, the invitation of Prof Stefan Litomericky, from the Czech Institute of Tuberculosis and Respiratory Diseases, to a WHO advisory group on ‘questions of effectivity of the support for health of the elderly’ to be held in Hamilton, Canada on 28–30 April 1986. No one from the USSR was invited. Letter no 2823/85 from the Embassy of the Czechoslovak Socialist Republic in Denmark to the WHO/HEE Office, 7 October 1985. WHO Archive, EURO-072, IRP/HEE/115-1.4.

57. Letter to the Members of the Expert Committee from June von Essen, Secretary, Health of the Elderly (HEE), 29 January 1988; WHO Archive, EURO-085, IRP/HEE 115.2.4, pp. 1–2; Telegram from WHO Copenhagen to Soiuzdravuvs, Moscow on 17 August 1987, WHO Archive EURO-090, IRP/HEE 120; Telex sent to ‘Kosenko’ at the Soiuzzdravuvs, Moscow, by David Macfadyen on 13 October 1987, WHO Archive EURO-085, IRP/HEE 115.2.4; Telegram sent by Kosenko to Macfadyen, 5 October 1987, WHO Archive, EURO-085 IRP/HEE/115.2.4; Euro Memorandum, ‘Expert Committee on Health of the Elderly, Geneva, 2–9 November 1987, Addendum II’, 31 July 1987, WHO Archive EURO-085, IRP/HEE 115.2.4.

58. WHO, ‘Special Programme for Research on Aging. Advisers and Staff. Programme content. Research management’, August 1987. WHO Archive, EURO-090, IRP/HEE 120, document pp. 1–2.

59. On Bezrukov’s return to Kyiv, see author's interview with Vladislav Bezrukov, Kyiv, September 2019; Letter from Bezrukov to I. N. Denisov, deputy minister of healthcare of the USSR, dated 16 September 1988, GARF f. 8009, op. 55, d. 79, l: 70.

60. Dr David M. Macfadyen, ‘Our Life in Our Times: Reflections on the Vienna International Plan of Action on Aging’, Paper prepared for the XI International Congress of EURAG, Rome, 6–9 May 1985, WHO Archive EURO-86, IRP/HEE 116.03, report p. 6.

61. Author's interview with Vladislav Bezrukov, Kyiv, September 2019; also see Sidorenko (n.d.).

62. Document ‘Purpose and Objectives of Expert Group Meeting on Long-Term Training and Education in Gerontology and Geriatrics’ 20–24 February 1989, Valleta, Malta. EURO-69, IRP/HEE/114.
